# Nebulized dexmedetomidine in the treatment of obstetric post-dural puncture headache: two case reports

**DOI:** 10.1186/s12871-025-02896-4

**Published:** 2025-01-11

**Authors:** Jeffrey Thomas, Leonard J. Soloniuk, Chris Mehdizadeh, Peter Cheng, Ashish Sinha

**Affiliations:** 1 Department of Anesthesiology and Perioperative Medicine, Riverside University Health Systems, Moreno Valley, CA USA; 2 Department of Anesthesiology, Loma Linda University Medical Center, Loma Linda, CA USA; 3 Department of Gynecology and Obstetrics, Loma Linda University Medical Center, Loma Linda, CA USA; 4 Department of Internal Medicine, University of California Riverside, Riverside, CA USA; 5 University of California Riverside School of Medicine, 900 University Ave, Riverside, CA 92521 USA; 6 New York Medical College at Saint Mary’s General Hospital, Passaic, NJ USA

**Keywords:** Obstetric post-dural puncture headache (PDPH), Obstetric anesthesia, Neuraxial anesthesia, Dexmedetomidine, Cesarean delivery, Case report

## Abstract

Post-dural puncture headache (PDPH) is a debilitating complication of neuraxial anesthesia, particularly prevalent in obstetric patients, usually characterized by a postural headache. PDPH is hypothesized to result from cerebrospinal fluid leakage through a dural puncture, triggering symptoms like neck stiffness and subjective hearing changes. While conservative measures are common for treatment, more refractory cases may require invasive interventions such as an epidural blood patch (EBP). Recent studies have shown promise in using nebulized dexmedetomidine (nDEX) for PDPH, offering a non-invasive alternative to EBP. Two case presentations illustrate the efficacy of nDEX in resolving PDPH symptoms rapidly and completely. These cases underscore the need for exploring novel therapeutic options, especially in obstetric patients where safe and prompt relief is essential for maternal and newborn well-being. While the EBP remains the gold standard, its limitations of accessibility and invasiveness highlight the significance of investigating alternatives like nDEX.

## Introduction

Post-dural puncture headache (PDPH), an incapacitating positional headache worsened by upright posture and relieved by supine positioning, remains a common complication of neuraxial anesthesia, especially in obstetric patients. The International Headache Society (IHS) defines PDPH as a postural headache occurring within five days of a lumbar puncture, and usually remits spontaneously within two weeks or after sealing of the leak with an autologous epidural blood patch (EBP) [1]. It is often accompanied by neck stiffness and/or cranial nerve dysfunction.

PDPH can cause significant clinical sequelae that leads to increased pain and hospital length of stay. One retrospective study of 2113 obstetric patients found that PDPH increased hospital length of stay by two days on average when compared to patients without PDPH [2]. 

PDPH is likely precipitated by continuous cerebrospinal fluid (CSF) leakage through the dural tear causing neural traction and pain from decreased CSF volume and pressure. As little as 10% volume loss of total CSF can trigger symptoms. Current mainstays of treatment include conservative measures like bed rest, caffeine, fluids, and analgesics. EBP, while effective, is an invasive option and may require the patient to return to the hospital. A case series of six patients was reported of administering nebulized dexmedetomidine (nDEX), 1 µg/kg, twice daily for three days to treat PDPH with promising results [3]. A single-center randomized, double-blind controlled study treated PDPH with nDEX, 1 µg/kg, twice daily for 72 h or until improvement in symptoms [4]. This study demonstrated significant reductions in visual analog pain scores (VAS) and Lybecker scores with nDEX. Here, we present two case presentations that illustrate the efficacy of nDEX in resolving PDPH symptoms rapidly and completely. Written consent was obtained from both patients involved, agreeing to the use their de-identified information in the preparation of this manuscript.

## Case Presentation 1

29-year-old G2P1 (Height 152.4 cm, BMI 28.24 kg/m^2^) at term with a history of diabetes, Class II obesity, and prior cesarean section under spinal anesthesia presented for scheduled repeat cesarean delivery. Multiple attempts were made to access the intrathecal space without success, and an attempt was made to access the subarachnoid space using a 17-gauge Tuohy needle as a guide for the spinal needle. An unintended dural puncture (UDP) occurred, and the patient developed signs and symptoms of a PDPH. Conservative management with bed rest, acetaminophen, hydrocodone, butalbital/caffeine, ibuprofen, and intravenous fluids was initiated without resolution of symptoms and minimal improvement in pain (VAS from 7 to 6) and functional status. Twenty-four hours after UDP, nebulization was performed using dexmedetomidine 1 µg/kg body weight, diluted out to 3 mL with preservative-free normal saline. The diluted dexmedetomidine solution was placed in the nebulizer (AirLife^®^ Misty Max 10™, Sun Med, Mexico) with an oxygen flow of 10 L/min until the solution was fully nebulized. A face mask or mouthpiece was used depending on the patient’s preference. Rapid and complete resolution of headache and pain (VAS = 0) after the single dose.

## Case Presentation 2

25-year-old G2P1001 (162.5 cm, 34.50 kg/m^2^) at 39w2d gestation without significant medical history presented with a spontaneous rupture of membranes. Epidural analgesia was planned. A 17-gauge 3.5-inch Tuohy needle was introduced at the L4-5 level using a midline approach and the epidural space was identified at a depth of 7 cm using a loss of resistance technique at first pass. No UDP was recognized. A 20-gauge catheter was placed without complications. A test dose of 3 mL of 1.5% lidocaine and epinephrine (5 µg/mL) was administered without side effects. Following initiation of programmed intermittent boluses, hypotensive episodes occurred, prompting the change in programming from intermittent 10 mL boluses every 40 min to 3 mL every 40 min.

She had a spontaneous vaginal delivery without complications and was discharged on postpartum day one. Two days later, the patient was readmitted to the hospital for a new-onset episodic 10/10 bifrontal headache that lasted for 30 min at a time, recurring every 3–4 h, and radiated to the neck/upper back, and is worsened with upright posture and improved in recumbent positioning. There was associated nausea, photophobia, unilateral arm numbness, tingling, and lightheadedness. Laboratory testing showed no abnormalities, and a tentative diagnosis of PDPH was made. Acetaminophen, ibuprofen, and oral caffeine pills were administered, and the patient was maintained in a recumbent position with little improvement. The patient was informed of the risks and benefits of EBP and she declined placement of an EBP. She was informed of the alternate treatment of nDEX with its potential risks and benefits. Nebulization was performed using dexmedetomidine 1 µg/kg body weight, diluted out to 3 mL with preservative-free normal saline. The diluted dexmedetomidine solution was placed in the nebulizer (AirLife^®^ Misty Max 10™, Sun Med, Mexico) with an oxygen flow of 10 L/min until the solution was fully nebulized. A face mask or mouthpiece was used depending on the patient’s preference. Immediate resolution of symptoms occurred with a single dose of nDEX, and the patient was discharged to home the same day.

## Discussion

The postpartum period is characterized by numerous changes such as irregular food intake, dehydration, and sleep deprivation – all factors that could contribute to a headache. Postpartum headache is common at 39% incidence, with only 4.7% of headaches being anesthesia related [5]. Since PDPH is a clinical diagnosis, identification and management requires careful clinical assessment to make the diagnosis.

An estimated 80% of women in developed countries receive epidural analgesia during labor as a safe and effective measure for providing relief of labor pain [6]. Risk factors for PDPH, like female gender, pregnant state, young age, anemia, as well as frequent use of spinal anesthesia for cesarean delivery render this population vulnerable to PDPH [3, 7]. Placement of an epidural catheter carries the risk of UDP and has an incidence of 1–6%.^8^ In obstetric anesthesia, UDP results in PDPH in up to 81–88% of UDPs [6–8]. 

Dexmedetomidine is a highly selective alpha-2 adrenoreceptor agonist commonly used for sedation and analgesia. It acts centrally to suppress neuronal firing and neurotransmitter release responsible for pain signaling. An additional benefit is cerebral vasoconstriction from alpha-2 stimulation, thereby reducing cerebral blood flow. The use of nDEX may serve as a useful adjunct in situations that require cerebral vasoconstriction with analgesia such as PDPH, as evidenced in these reported cases.

Several studies have been performed comparing the efficacy of nDEX versus nebulized neostigmine/atropine, nebulized fentanyl and placebo (nebulized saline) in obstetric patients with PDPH (Table 1) [4, 9, 10]. Kumar and associates demonstrated in a double-blinded randomized study that pain scores in the nDEX group were significantly lower than in the nebulized fentanyl and placebo groups [9]. While Soliman and associates demonstrated in a randomized double-blind controlled study that VAS was significantly decreased in nDEX, and neostigmine/atropine groups compared to the control group at six hours [10]. 

**Table 1:** Review of current literature on nebulized dexmedetomidine for PDPH

Study	Design	No. of participants	Clinical question	Conclusion
Kumar et al. (2024) [9]	Prospective	90	To compare nDEX versus nebulized fentanyl for the treatment of PDPH in parturients after caesarean section under spinal anesthesia.	The pain scores up to 72 h following nebulization were significantly lower in the DEX group in comparison to the fentanyl group and control group (*P* < 0.001). Overall additional analgesic requirement in the DEX group was significantly lower in comparison to the other groups (*P* < 0.001).
Mowafy et al. (2021) [4]	Prospective	43	This study aimed to test the effectiveness of nDEX for conservative management of PDPH and evaluate its cerebral hemodynamic effects transcranial Doppler.	VAS and Lybecker scores were significantly lower in the DEX group. The middle cerebral artery mean flow velocity was significantly lower, and the pulsatility index was considerably higher after nDEX compared to placebo.
Soliman et al. (2023) [10]	Prospective	90	To compare the effectiveness of nDEX versus neostigmine/atropine in the conservative management of PDPH.	VAS was significantly decreased in DEX, and neostigmine/atropine groups compared to the control group at six hours. No patients in the DEX group, but one patient in the neostigmine/atropine group and seven patients in the control group needed an epidural blood patch.

Dexmedetomidine (DEX); Nebulized dexmedetomidine (nDEX); Post dural puncture headache (PDPH); Visual Analog Scale (VAS)

While the EBP remains the gold standard for definitive treatment, it does require specialized training and equipment, typically administered by an anesthesiologist or pain management specialist. The patient may need to be positioned carefully, and sterile techniques must be followed to minimize the risk of infection. Risk versus benefit of EBP must be considered given reports of efficacy ranging from 61 to 73% to achieve partial relief of headache and 10–32% to achieve complete relief of headache [11]. Additionally, the procedure is usually performed in a hospital or outpatient setting, which may limit its accessibility for some patients.

## Conclusion

These cases highlight the importance of evaluating novel therapeutic options for PDPH, particularly in obstetric patients where rapid symptom relief is crucial for maternal well-being and postpartum care. Thereby, alternatives to EBP such as nDEX may provide a non-invasive, highly accessible means of treating patients, especially in time and resource-constrained settings without the access of trained anesthesiologists to administer an EBP. Further research into the efficacy and safety of nDEX in managing PDPH is warranted, offering the potential for a non-invasive and convenient alternative for patients experiencing this debilitating complication.

## Data Availability

The data of the two patient cases supporting the conclusions of this article are included within the article in Case Description. No public dataset or database was utilized.

## Glossary of terms

PDPH: Post-dural puncture headache.
UDP: Unintentional Dural Puncture.
nDEX: Nebulized Dexmedetomidine.
EBP: Epidural Blood Patch.
BMI: Body Mass Index.
CSF: Cerebrospinal Fluid.
VAS: Visual Analog Pain Scores.
